# Fibrin Strands Will Grow from Soluble Fibrin and Hang Up in an In Vitro Microcirculatory Viscoelastic Model: Is This a Major Cause of COVID-19 Associated Coagulopathy?

**DOI:** 10.3390/jcm11082084

**Published:** 2022-04-07

**Authors:** Brian S. Bull, Karen L. Hay, Paul C. Herrmann

**Affiliations:** Department of Pathology and Human Anatomy, Loma Linda University, Loma Linda, CA 92354, USA; yahk@verizon.net (B.S.B.); khay@llu.edu (K.L.H.)

**Keywords:** COVID-19, fibrin, fibrinogen, fibrinolysis, fibrinolytic shutdown, rotational thromboelastometry (ROTEM), soluble fibrin monomer complex (SFMC), thromboelastography (TEG), thrombosis, viscoelastic

## Abstract

Viscoelastic testing (VET) by both TEG and ROTEM has demonstrated hypercoagulability early in corona virus disease 2019 (COVID-19) associated coagulopathy (CAC). Additional VET studies demonstrated fibrinolytic shutdown late in a majority of severely ill COVID-19 patients with an associated elevation of d-dimer. Elevated d-dimer confirms that coagulation, followed by fibrinolysis, has occurred. These findings imply that, during CAC, three enzymes—thrombin, Factor XIIIa and plasmin—must have acted in sequence. However, limitations in standard VET analyses preclude exploration of the earliest phases of clot induction, as well as clot formation and clot dissolution in flowing blood. Herein, we describe a novel method illuminating aspects of this unexplored area. In addition, we created an in vitro blood flow model in which the interactions of thrombin, Factor XIII and plasmin with fibrinogen can be studied, allowing the determination of soluble fibrin (SF), the highly unstable form of fibrin that precedes the appearance of a visible clot. This model allows the determination of the SF level at which fibrin microclots begin to form.

## 1. Introduction

There is little question that clotting, in some form, accompanies severe COVID-19 pneumonia [1,2,3,4,5]. The d-dimer level in such patients is routinely elevated—yet gross clots are detected in only a minority. What accounts for the d-dimer elevation in the remaining patients? Furthermore, does that elevation truly reflect the presence of widespread intravascular clotting? If that is the case, then the pathophysiology of severe pneumonia is primarily that of a clotting disorder in many, perhaps most, intensive care unit (ICU) COVID-19 patients.

Currently, the most commonly used coagulation test methods available are not useful for detecting hypo and hypercoagulopathic states beyond those associated with severe factor deficiencies or strong clotting function inhibitors. Consequently, it is very difficult to provide laboratory guidance in a number of clinical situations, such as liver transplantation, cardiac trauma and obstetrics, and particularly with regard to the care of the COVID-19 patient. Viscoelastic testing (VET) methods, such as thromboelastography (TEG) and rotational thromboelastometry (ROTEM) provide deeper insight into these subtle coagulopathic changes and have demonstrated that COVID-19 patients develop a propensity for hypercoagulation [6,7,8,9,10,11,12,13,14]. Herein, we provide structural images of what may connect the COVID-19 patient’s hypercoagulopathy with pneumonia and other organ system failure. In addition, we demonstrate a method of testing for an early risk of hypercoagulability based on the pre-clot formation of soluble fibrin that provides information complementary to that obtained through VET, providing an early-warning system and allowing appropriate clinical intervention.

The coagulopathic changes in COVID-19 associated coagulopathy (CAC) [15,16] are not identical to those of disseminated intravascular coagulation (DIC). However, some variant of intravascular clotting must be occurring, as evidenced by elevated d-dimer levels seen in a majority of COVID-19 patients upon admission [17], and in virtually all ICU COVID-19 patients. Elevated d-dimer levels are associated with disease severity and adverse outcomes, including mortality, in COVID-19 patients [17,18]. d-dimer elevation confirms that Factor XIII-stabilized clots (Factor XIII crosslinked protofibrils or larger) have formed, and have subsequently undergone fibrinolysis [19]. It follows that un-crosslinked fibrin protofibrils and even earlier fibrin oligomers and monomers must have been present prior to the appearance of the Factor XIII-stabilized clots.

The final step in the clotting process is the conversion of fibrinogen to fibrin. Both the fibrinogen quantity and quality can affect the structure of the resulting fibrin clot. [20,21] Thrombin cleaves fibrinopeptides A and B (FpA, FpB) from nearby fibrinogen molecules, exposing active binding sites. Each modified fibrinogen molecule is now known as a fibrin monomer. The active sites on these monomers rapidly link with always-active complementary sites present on other fibrin monomers (forming oligomers of various sizes)—or they link with the always-active complementary sites which are also present on native fibrinogen. Early in the clotting process, with native fibrinogen in vast excess, most monomers and oligomers bind to intact fibrinogen and are carried throughout the circulation in a soluble form. These early “soluble” forms of fibrin have been referred to as soluble fibrin monomer complexes (SFMC) [22] or simply soluble fibrin (SF). Elevated SF levels are an early indicator that the clotting pathway has been activated and the process of clot formation has begun. If thrombin generation continues, monomer and oligomer numbers increase and SF levels rise. As oligomers grow in length, fibrin protofibrils form. Once the protofibrils reach ~20–25 monomers (0.5–0.6 µm) in length or longer, they will become insoluble [23,24], though still invisible to the naked eye. With further growth, they are easily visible in wet preparations under light microscopy—first as individual fibrin strands and, shortly thereafter, as larger fibrin clots. Protofibrils and larger fibrin polymers are stabilized (made resistant to lysis) when Factor XIIIa catalyzes covalent crosslinking between the D-domains of adjacent fibrin molecules [25].

At virtually the same time that thrombin initiates the clotting process, it also initiates fibrinolysis through the conversion of plasminogen to plasmin. The regulation of fibrinolysis is complex. Tissue plasminogen activator (t-PA) and plasminogen bind to the developing fibrin, leading to the conversion of the proenzyme plasminogen into plasmin. Intriguingly, both t-PA and plasminogen bind to fibrin, but not appreciably to fibrinogen. [26,27] Plasmin-initiated lysis of potentially all forms of fibrin—as well as intact fibrinogen, depending on the circumstances—ensues. Variously-sized fibrin(ogen) degradation products (FDP) are the result. However, any crosslinked D-domains are resistant to lysis, giving rise to degradation products with now-exposed D-D neoantigenic determinants. These antigenic determinants can be used as a tool for immunodiagnostic quantification of the so-called ‘d-dimer’ [28]. All d-dimer tests utilize monoclonal antibodies targeting various epitopes on the neoantigen [29], so are specific for the lysis of stabilized clots—clots in which adjacent fibrin strands have been covalently crosslinked.

By giving an early alert that the clotting process is ramping up, quantifying SF levels might well prove useful in guiding the optimum therapy for patients with coagulopathy, in general. It might also clarify some of the puzzling aspects of COVID-19 pathophysiology. In COVID-19, clotting followed by lysis of those clots happens in virtually all ICU COVID-19 patients, as evidenced by the markedly elevated d-dimer levels previously noted [17,18]. A determination of the SF level at which the first clots appear, as well as how long such clots persist in normal blood, would be highly informative.

Protamine sulfate solutions have long been known to precipitate soluble fibrin and other proteins [30,31]. We have developed a protamine sulfate-based method to quantify SF, based on the time it takes under controlled conditions for such precipitates to reach a predetermined size. The methodology involved in measuring SF levels in normal and pathological blood samples has been comprehensively detailed [32] and is briefly described below.

To further pursue an understanding of the early pivotal processes of clot induction and clot formation, we constructed a model system that—when exposed to elevated SF levels similar to those measured in COVID-19 patients—would support microclot formation, permitting the recovery of microclots for morphological investigations.

## 2. Materials and Methods

### 2.1. Measurement of Soluble Fibrin

The assay measuring SF levels was previously developed in our laboratory under the FDA rules for laboratory-developed tests (LDT) [33]. We developed the Rapid SF (Soluble Fibrin) assay [32] to address surgical needs during liver transplantation. Increasing SF levels provided an early warning to the liver transplant surgeons that DIC was likely underway.

The Rapid SF assay utilizes disposable glass test tubes, each containing 450 µL of protamine sulphate reagent (0.04 mg/mL in 0.9% saline) to which 150 µL of citrated whole blood is added. During the assay, the contents of the 12 × 75 mm tubes are exposed to a complex rolling and rocking motion to ensure that all SF molecules, as they emerge from the solution, will agglomerate into accumulations of precipitated fibrin. The size of these fibrin agglomerations can be determined visually as the entire system is illuminated from above and examined through an angled mirror, from below. The time-to-endpoint, reflective of the concentration of these molecules, is reached when the fibrin agglomerations reach 0.5 mm in diameter. SF levels—in soluble fibrin units (SFU)—are calculated as follows:

$$\mathrm{SFU}\;(\sec^{-1})=\frac{700}{\mathrm{SF}\;\mathrm{time}\;\left(\sec\right)}$$

The complex motion to which the tube is subjected is composed of a rolling motion of 15 rpm combined with a rocking motion of +4 to −2 degrees at a rate of 15 cycles/min.

We employed this Rapid SF assay to measure SF levels in the model system. For each of the in vitro experiments, citrated blood samples (1-part 3.2% sodium citrate +9-parts blood) were drawn from consented normal donors via phlebotomy using a butterfly after first withdrawing and discarding approximately 2 mL of blood.

**Figure 1:**
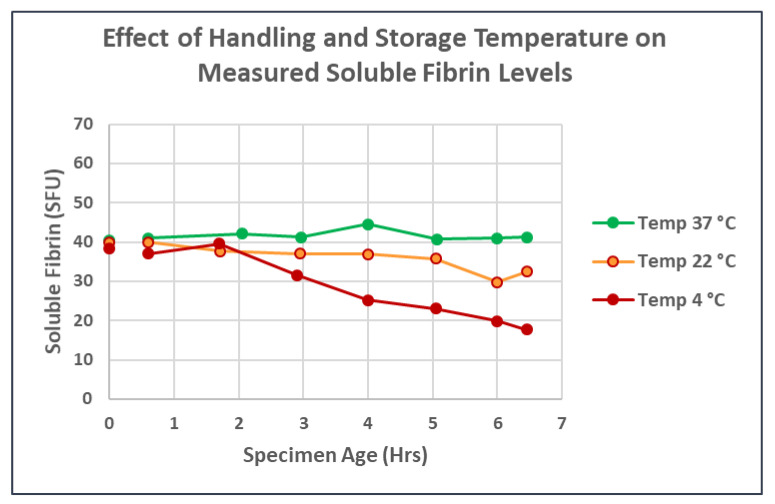
Blood was drawn from a normal donor and treated with thrombin, as described in Section 2.2.2, to achieve an SF of approximately 40 SFU. The treated blood was then each split into three syringes for storage at the three target temperatures (37 °C, 22 °C and 4 °C). Each syringe was initially tested (time 0) then all were placed at their respective temperature storage locations. At periodic intervals, each syringe was removed from storage, mixed, and a small portion removed and rewarmed to 37 °C for SF testing. Syringes were immediately returned to their designated temperatures. Samples with elevated SF that were maintained at 37 °C remained stable for over 6 h. However, at both room and refrigeration temperatures, measured SF levels dropped over time. In our experience, samples with SF levels greater than 50–60 SFU show precipitous drops in measured SF when cooled to 4 °C, with measured values plummeting into even the normal range. In contrast, SF values in the normal range show minimal change under the same conditions.

The stability of SF in blood samples is extremely temperature sensitive, and it is critically important that blood samples drawn for SF testing be maintained at 37 °C at all time points, from blood collection through to test completion. Any cooling of the blood will induce some degree of gelation and precipitation of the SF. When this happens, it is impossible to solubilize the precipitate by rewarming. Testing of the rewarmed sample will potentially result in extreme underestimates of the true SF level. The degree of underestimation depends on both the true SF levels at the time of drawing and the temperature to which the blood is cooled (Figure 1). There can be minimal effect in the normal range, but at markedly elevated SF levels, samples allowed to cool to room temperature, then rewarmed, may underestimate the true level by as much as 50–60%. Refrigerating samples can reduce the measured SF by 75% or more, on occasion dropping measured values back down into the “normal” range.

### 2.2. In Vitro Model System for Soluble Fibrin Production and Measurement

#### 2.2.1. Thrombin Preparation

Stock thrombin solutions (100 or 200 NIH U/mL) (equivalent to 100 or 200 IU/mL) [34] were prepared by reconstituting a vial of commercial lyophilized thrombin with sufficient volume to give the final desired concentration. We used thrombin, topical (Bovine origin), and USP (Jones Pharma Inc., St. Louis, MO, USA). Fill to half volume with Owren’s Veronal Buffer (Dade Behring, Inc., Deerfield, IL, USA), mix gently to achieve a full solution, then fill to the desired volume with glycerol. Aliquots were maintained at −20 °C in plastic vials until use. Working solutions were prepared by thawing an aliquot and diluting it 1:10 with normal saline to achieve a working concentration of 0.01 or 0.02 IU/µL (the currently recognized WHO International Standard for thrombin is expressed in international units (IU) rather than NIH Units (NIHU). Some of our thrombin sources were labeled using NIHU, others using IU, though the numbers themselves remain the same. For consistency, thrombin concentrations will be reported throughout the remainder of this paper in the IU format). Working solutions were prepared fresh daily and were maintained in plastic vials at room temperature while in use.

#### 2.2.2. General Method for Soluble Fibrin Production In Vitro

Blood samples for the in vitro experiments were provided by consented normal donors. Blood was maintained at 37 ± 1 °C throughout the entire subsequent process using temperature-controlled chambers, heat blocks or testing equipment, as needed. A small portion of the fibrinogen in anticoagulated whole blood was rendered into stable SF when minute quantities of the above thrombin were repetitively introduced into rapidly moving citrated whole blood—blood that was being rapidly swirled by hand around the base of a sterile, plastic specimen cup—during and for 60 s after each thrombin addition. Platelets were not inhibited. Repeated doses of 5–15 µL of working thrombin solution (0.01 IU/µL) added in this way to ~30 mL citrated blood gradually raised the level of SF in a stepwise fashion to a maximum of ~60 SFU, though higher levels could be transiently measured. (Using either larger thrombin volumes at each step or more concentrated thrombin tended to decrease the maximum SF value achieved while more quickly initiating grossly visible clotting.) A level of approximately 50–60 SFU appeared to be the maximum semi-stable level obtainable. If additional microliter aliquots of thrombin were added, SF levels did not increase further (except very transiently). Rather, monomers/oligomers departed the soluble phase, forming microthrombi at the point when SF levels began to plateau. With further thrombin additions, increasingly large, visible macroclots would form. At no point were the blood samples recalcified. Soluble fibrin testing was repeated 3–4 h later on residual samples that were maintained unmixed at 37 °C, then samples were spun down for plasma harvesting and subsequent measurement of fibrinogen levels.

#### 2.2.3. Precipitation of SF: Production, Recovery and Photography of Microclots

Whole blood samples with SF levels of ~50 and higher were prepared as above, with several minor modifications. Since prostaglandins synthesized by healthy normal endothelium maintain circulating platelets in a quiescent condition, for these experiments involving the production and recovery of the fibrin fibrils and fibrin microclots, platelets were inhibited by the addition of the synthetic prostaglandin Iloprost (Berlex Laboratories, Hanover, NJ, USA) at a final nominal concentration of 0.15–0.2 µG/mL blood. A 20 mL volume of citrated blood was drawn and baseline levels of SF were measured on healthy, normal adult donors, as described above. Then, increasing levels of SF were generated by the repetitive addition of 10 µL aliquots of working thrombin (0.02 IU/µL) as the blood was rapidly swirled by hand around the base of the ~50 mm sterile plastic cup maintained at 37 °C. Between thrombin additions and otherwise throughout the experiments, the blood was continuously swirled in a temperature-controlled container at ~90 rpm by an orbital rotator (“VEVOR Orbital Rotator Shaker: Adjustable Speed at 0-210 RPM, Digital Orbital Shaker 0–15 min, Orbital Oscillator with 12.4 × 8.6 Inch Working Platform, Orbit Shaker for Chemical, Biological Laboratories” from Amazon Shopping). SF levels were measured 60 s following each thrombin addition.

Once the SF levels were raised to 50–60 SFU, the addition of as little as 5 µL of the working thrombin (0.02 IU/µL) rendered the soluble fibrin suspensions unstable and microclot formation would commence as the SF level began to plateau. Since the SF levels in this range had been seen in a number of severely ill COVID-19 ICU patients who had been clinically monitored with SF testing, we thought it appropriate to explore the relevance of these markedly elevated SF levels to COVID-19 pathophysiology.

**Figure 2:**
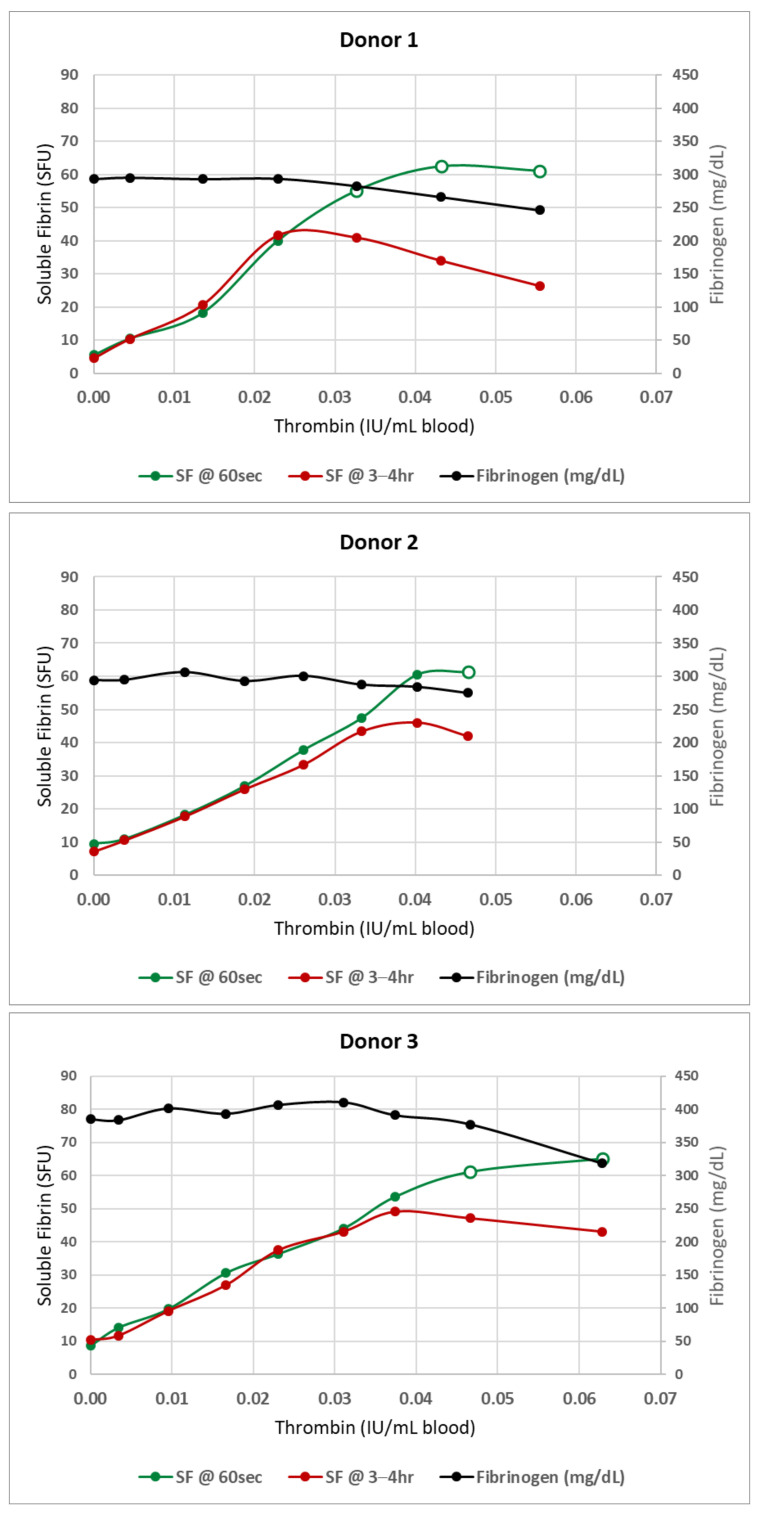
Plots similar to one previously published [32] showing the initial linear phase of stable soluble fibrin formation followed by a gradual bending of the curve (open circles) at which point soluble fibrin levels are no longer stable and insoluble microclots begin to form. Baseline SF levels were determined for each donor. Then increasing levels of SF were generated by incremental addition of minute volumes (5–15 µL) of thrombin to ~30 mL of rapidly-swirling normal donor blood. After each addition, the blood was manually swirled for an additional 60 s, then the SF level was measured. Prior to addition of the next increment of thrombin, an aliquot of blood was removed and maintained unmixed at 37 °C for SF retesting and fibrinogen determination at 3–4 h. SF increased linearly with each addition of thrombin until SF levels reached 40–60 SFU. Additional thrombin beyond this point increased the SF somewhat, but only temporarily, and the blood became increasingly ‘sticky’ with occasional fibrin strands noted. When retested at 3–4 h, the SF levels seen during the linear phase were maintained; but beyond this point, fibrin strands and clots were present in increasing numbers, and both measured fibrinogen and soluble fibrin levels began to drop, reflecting the conversion of fibrinogen—via soluble fibrin—into insoluble fibrin clot. The beginning of SF plateau (region of open circles) became the trigger for preparing filters in the subsequent experiments.

The beginning of SF plateau (open circles, Figure 2) was the trigger for starting the filtration steps. The generated in vitro whole blood samples containing these markedly elevated SF levels were passed through 13 mm diameter disks placed in a 13 mm Swinnex polypropylene filter holder. The disks were prepared from sheets of 35 micron nylon mesh (“35 Micron Nylon Mesh Filter Woven Mesh Sheet Off-White Polyester Food Grade” from Amazon Shopping). Blood was passed through at a flow rate of 1 mL/min followed by 3 mL of normal saline wash at the same velocity to remove cellular elements that were not physically attached to the developing microclots.

The nylon grids with microclots affixed to their surface were then fixed with 2% glutaraldehyde in cacodylate buffer and stained with Sypro Ruby Biofilm Matrix stain. The stained grids, either intact or disrupted so as to release the microclots, were photographed on a Zeiss LSM 710 confocal microscope equipped with an Argon multiline laser. The excitation wavelength was 514 nm. The emission signal was recorded from 463–735 nm. All photographs were taken through an EC Plan NEO 10X 0.3 NA or through a Plan-APO 20X 0.8 NA objective. Image J software was utilized to adjust the picture outlines, insert scale markers and crop the images when necessary.

The earliest detectable fibrin fibrils were ~5.0 µm long—the smallest that could be visualized microscopically in wet preparations. Further disks were prepared at approximately 5 min intervals during the early phases of fibril lengthening, stretching to 10–15 min intervals later as the microclots grew from single fibrils to multiple fibrils. Later disks often showed gradually decreasing microclot formation or total absence of microclots, as long as no further thrombin aliquots were introduced into the swirling blood. This raised the possibility that fibrinolysis was taking place, getting the upper hand over the clotting process. If, however, further aliquots of thrombin were introduced, the process of microclot formation continued.

## 3. Results

The introduction of sequential minuscule amounts of thrombin to a starting volume of 20–30 mL of whole blood rapidly raised the SF in the model systems to the elevated levels seen in severe COVID-19 pneumonia without producing any clots visible to the unaided eye—as long as the thrombin aliquots were added to rapidly-swirling blood. If the thrombin aliquots were added to static blood (data not shown), visible macroclots rapidly appeared.) The final thrombin concentration (calculated from the total thrombin added to this point) was typically <0.07 IU/mL of whole blood. However, given the fact that each dose of infused thrombin was rapidly and almost instantly diluted (by as much as 1:3000) by swirling, the active thrombin remaining was almost certainly near zero. Antithrombins present in normal blood would have neutralized any residual traces of thrombin within seconds. Figure 2 shows the linear relationship between the SF level and thrombin concentration during the initial phase of SF generation. However, when SF levels approached 50 SFU, the curves began to flatten (open circles). At this point, fibrin oligomers were typically large enough that microclots were beginning to form and precipitate out of solution—effectively lowering the SF level. The SF measurements obtained on the same blood samples 3–4 h later remained unchanged in the linear phase—but showed a marked drop in SF in the plateau region, reflective of continued, progressive clotting over time. The fibrinogen levels began to drop at the same point, reflecting the removal of precipitated clots when plasma was harvested by centrifugation for subsequent testing.

In the model filter system, at 5–7 times the upper limit of normal (levels similar to those maintained over several days in some severely ill COVID-19 patients), [35] SF began precipitating as fibrils only a few microns long and less than a micron wide. With no further addition of thrombin once the microclots initially formed, they grew rapidly until they partially or completely occluded the 350 µm^2^ channels in the sieve. Longer microfibrils, then accumulations of microfibrils, then microclots continued to form. Microclots of this size were individually invisible to the naked eye, and—in patients—would be clinically invisible. Over time, the microclot concentration noted on sequential disks tended to decrease. In some donors, they disappeared within 15 min or so; in others, they persisted for 30–60 min. In addition, some donors seemed altogether resistant to clot formation, with no clots ever forming, raising the possibility that ongoing and very active lysis successfully overcame the development of any clots. The varied persistence of clot generation between different blood donors may indicate that the balance between clotting and fibrinolysis might vary enormously between different persons.

Individual fibrin strands and early microclots were recovered only after the SF levels exceeded at least 50 SFU. In some normal blood donors, SF levels as high as 60–70 SFU were required. The most reliable indicator that microclots had begun to form was the flattening of the curve relating SFU levels to the amount of thrombin that had been infused (Figure 1). It is surprising that such simple maneuvers as adding thrombin in tiny amounts into rapidly moving blood—thus simultaneously diluting and neutralizing it—will so dramatically alter the physical form of the fibrin that is produced.

The sieve material presented rows of alternate elevations and depressions, each containing four 10 × 35 µm (surface area of each pore) rectangular sieve-pores. Adjacent 350 µm^2^ sieve-pores were thus separated by a nylon fiber 35 µm in diameter. The major portion of each 2 mL of sieved blood traversed these rectangular pores. As a consequence, it was here that the individual fibrils first draped themselves over the nylon fibers that separated adjacent sieve-pores. It was also in these regions that the microclots grew most rapidly in size during the filtration process as additional SF molecules precipitated onto the already formed and arrested microfibrils, adding to the developing microclot.

**Figure 3:**
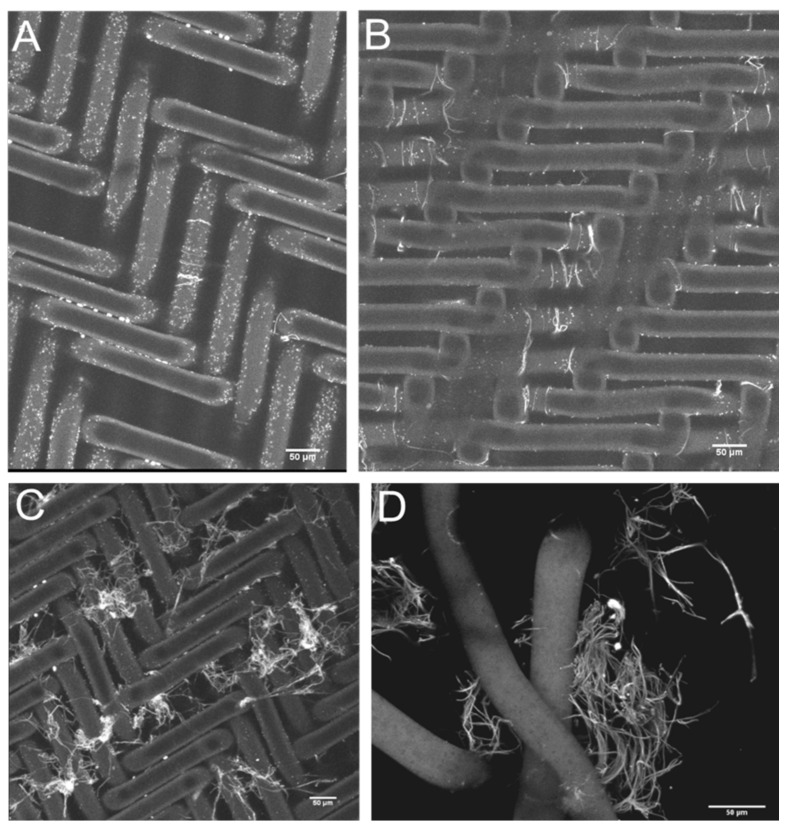
The sieve fabric is woven in a herringbone pattern from 35 µm diameter nylon filaments. Images (**A**–**C**) are 3D reconstructed, maximum intensity, images collected with a 20× air objective of 6 z-stacks (1.3 µm slices) of a centered tile grid 2 × 2 with 10% overlap. The herringbone-pattern nylon grid was stained with Sypro Ruby Biofilm Matrix stain. (**A**) The pores in the sieve are nominally 10 × 35 µm and groups of four pores in a depression in the fabric are in the base of each of the four darker rectangular areas of the grid. The small white fibrils are the earliest indication of SF having transformed into insoluble fibrin microfibrils. The tiny white dots also precipitate along with the fibrin fibrils and disappear later as fibrinolysis commences. They possibly represent amyloid type fibrin rather than the properly polymerized variety. (**B**) Approximately ten minutes after the start of fibril formation, several longer fibrils are draped over the nylon filaments that bridge between adjacent sieve-pores. (**C**) Approximately twenty minutes into the process of microclot formation, small early microclots are now evident. (**D**) The woven nylon filament grid has been disrupted and the forming microclots have been recovered, fixed and stained. Three torn nylon filaments from the disrupted sieve can be seen in the foreground. 3D reconstructed, maximum intensity images collected with a 10× air objective of 11 z-stacks (6.62 µm slices). Sypro Ruby Biofilm Matrix stain.

**Figure 4:**
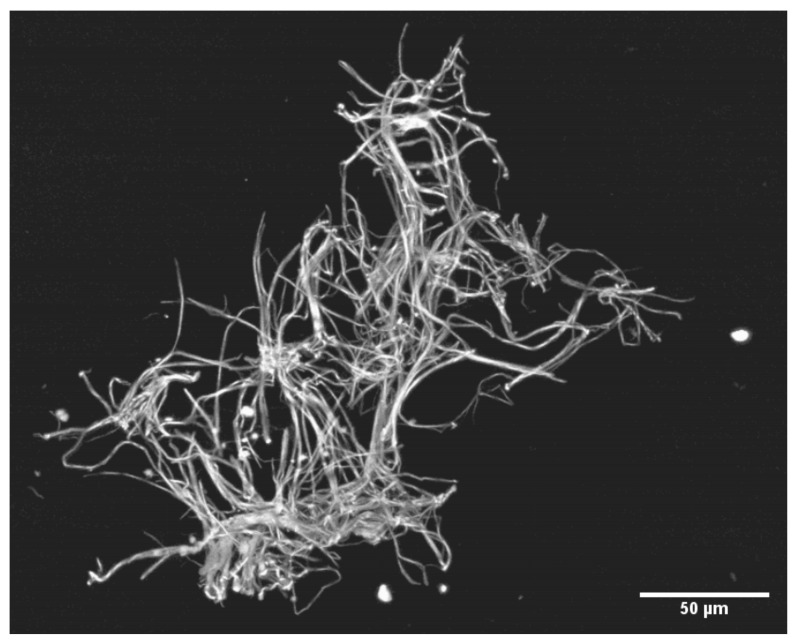
A larger microclot. Three-dimensionally reconstructed; maximum intensity images of 11 z-stacks (6.62 µm slices). Sypro Ruby Biofilm Matrix stain, 20× air objective.

**Figure 5:**
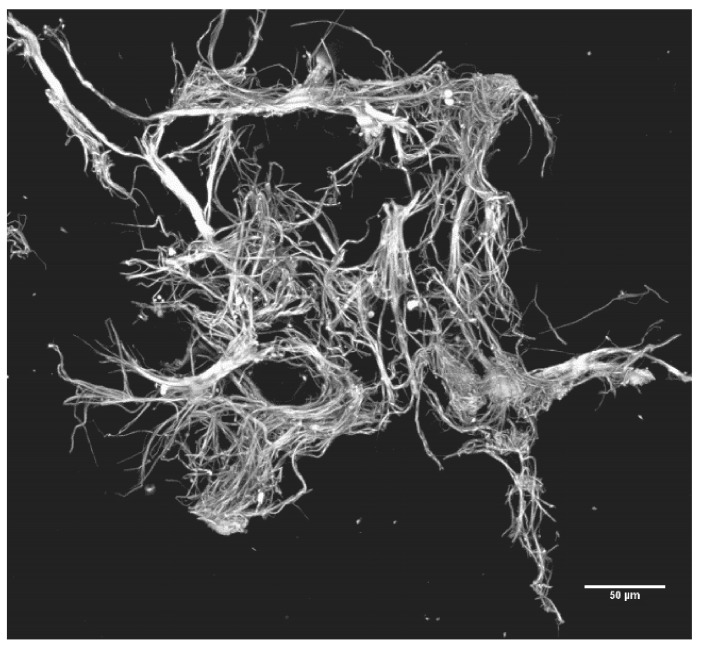
A still larger microclot. Much of the bulk of this clot and that shown in Figure 3 likely formed in the swirling blood rather than on the sieve surface. However, microclots of this size grow even larger when immobilized on the sieve surface as SFMC passing over and through the immobilized clots cause them to rapidly increase in size. Maximum intensity images of 13 z-stacks (6.62 µm slices). Sypro Ruby Biofilm Matrix stain 20× air objective.

In Figure 3, the developing microclots have been fixed in situ and then detached from the nylon grid by disrupting the woven grid fabric. Figure 4 and Figure 5 depict the larger microclots that form in the swirling blood as smaller microclots encounter additional soluble fibrin molecules and continue to enlarge. It is notable that red cells do not adhere to these clots, although the clots have formed in whole blood. This unusual outcome (for whole blood clots) is likely due to the fact that these fibrin fibers lengthened primarily at the fiber ends and branched infrequently. If they lengthen slowly enough, red cells will not be enmeshed and trapped in the interstices of the developing microclot.

## 4. Conclusions

The Rapid SF assay provides information otherwise unavailable by standard laboratory clotting studies. Increasing SF levels are an early-warning indicator that in vivo thrombin generation has begun, well before there is any other laboratory or clinical evidence of hypercoagulability. It thus informs our understanding of the very early steps in the sequence of events within the clotting process and, hence, provides clinical insight into patient coagulation status. Although our SF discussion in this paper revolves primarily around the thrombotic tendency in COVID-19 patients, it is in fact valuable in any situation involving increased thrombotic risk (DIC, liver transplant, patients with ventricular assist devices, trauma, sepsis, pregnancy complications, snakebite, etc.).

As a measure of the earliest phase of clotting—the formation of soluble fibrin monomers and oligomers—the SF assay provides an additional piece of information that can be useful in determining when anticoagulation therapy may best be started. By starting anticoagulant therapy and then monitoring the patient through the additional highly sensitive VET methods that likely inform anticoagulation dosing as well as providing a platform for the monitoring of therapeutic levels of anticoagulation, it may be possible to avoid the dual harms of too late a start to therapy on the one extreme, balanced against over-anticoagulating patients that would be better served left in homeostasis, on the other. We believe the complementarity of these testing methods to be superb.

This assay for soluble fibrin provides results in real-time, making it possible for the blood specimens to be analyzed immediately without further manipulation upon arrival in the laboratory—as long as the samples have at all times been maintained at 37 °C. At all measured levels, SF has proven to be temperature sensitive, with the sensitivity increasing as the true SF level rises. While samples with normal SF levels are minimally affected, those with the highest levels are dramatically affected. Allowing patient specimens to cool to room temperature between acquisition and analysis decreases the measured levels of SFMC substantially. The original levels cannot be recovered by rewarming.

This temperature sensitivity might possibly account for some of the bizarre skin manifestations that have been reported in COVID-19 patients [36,37]. It also underscores that reliable and reproducible assays of SF blood levels are likely to be possible only if 37 °C is maintained from specimen acquisition throughout specimen analysis.

If the addition of minute amounts of thrombin—followed instantaneously by dilution and neutralization of the thrombin by natural antithrombins—successfully produces SF molecules that are similar to the SF molecules produced in the circulatory system of COVID-19 patients, some interesting speculations are present. The clots that form on the nylon mesh increase rapidly in size as additional SF-rich blood flows over and through the developing fibrin meshwork. It is tempting to speculate that a similar process is occurring in both the systemic and the pulmonary circulation of COVID-19 patients. This is an unusual coagulopathy in the relationship and propinquity of thrombin to clot formation. Here, whatever thrombin was present when SF molecules were originally generated, that thrombin has long since been dissipated by both massive dilutions and by the antithrombins present in normal blood. Therefore, further growth of these microclots is not supported by SF newly generated by thrombin in the immediate environment. In a static assay system, the microclots can only grow as rapidly as the diffusion of already-formed SFMC allows. However, this would be quite limiting and would be expected to allow for only very tiny microclots to form. In a flowing system (as in the model described here) this limitation no longer exists. Further SF molecules and fibrin fibrils will be transported to any already-arrested fibrin fibrils and add to the growing accumulation. In the model system, it is often the case that the earliest stage of microclot formation consists of a single or only a few fibrin fibrils draped on one of the much larger nylon fibers separating two adjacent 10 × 35 µm pores in the woven filter mesh. If this is indeed also happening in the microcirculation of COVID-19 patients, then microclot formation would largely occur where an arrested fibrin fibril nidus is being fed by SF-rich flowing blood. Furthermore, growth would stop when flow ceased as soon as the microclot grew sufficiently large to occlude one of the vessels below the dividing point in the vessel where the original clot started to form.

What might be the potential for harm if a similar process is occurring in the microcirculation? If, in the circulation of patients with COVID-19 pneumonia or other coagulopathy, microclots are forming and randomly occluding microcirculatory vessels and the occlusions are only temporary, little damage may result, as normal fibrinolysis will rapidly remove such microclots. However, if the occlusions persist for minutes to hours—as they very well may during fibrinolysis shutdown—small portions of downstream organs and organ systems will undergo anoxemic coagulation necrosis. Miniscule thrombi can be readily identified in the majority of ventilator-dependent COVID-19 patients if the microcirculation is examined by supravital capillaroscopy [38]. It is possible that this process, occurring in widely separated micro-sites throughout the body, is responsible for the tissue damage that underlies both the protean manifestations of COVID-19 pneumonia and also many of the puzzling ‘Long-COVID’ symptoms.

In a previous paper [35], it was proposed that 7–10 days into a COVID-19 infection, a “macrophage attack” heralds the appearance of elevated levels of SF in the bloodstream. This development indicates that the COVID-19 case has transformed into a whole-body clotting disorder (with, of course, continuing viral pneumonitis). If indeed, this is the significance of high levels of SF in the circulation, then this development affects both the manner in which this polyphasic disorder is optimally treated as well as the way in which therapeutic advances are best evaluated in the future. Clearly, an understanding of the pathophysiology of this disorder is a first step toward the development of effective treatment.

Newer therapies, novel medicinal agents, etc. would be most beneficial if administered only during the phase of the disease that they were designed to treat. Failure to do so could easily obscure the real benefits of a therapeutic agent by the inclusion of patients suffering from a phase of COVID-19 that was not susceptible to the medicinal agent under study. If much of the pathophysiology of COVID-19 reflects the sequelae of transient microclots causing patches of coagulation necrosis affecting all organ systems, and if such patients are included in the study group, the beneficial effects of antivirals will be seriously underestimated. For similar reasons, the benefits of convalescent plasma will also be underestimated (it would be expected to dampen down the viral replication phase but have only minimal effect on the clotting phase).

On the brighter side, if the increased lethality of COVID-19 is, to a significant extent, tied up with its transformation into an unusual DIC variant then, perforce, there exist several therapeutic avenues that are presently incompletely explored. In the first few days, attempts to slow down viral replication would take pride of place, for if the viral load is minimized, it is less likely that the macrophages will gather. Agents that directly attack the virus obviously show promise, but so would agents that increase the nonspecific anti-viral response. Deficiencies of vitamin D and zinc are common in the elderly and increase the lethality of viral infections. These deficiencies could be readily addressed in vulnerable populations. At the very least, some of the molecules that attract macrophages to the site of virally damaged cells have been identified [39]. The I-SPY-COVID-19 Trial is a research trial designed to investigate an adaptive platform approach to reduce mortality and ventilator requirements for critically ill COVID-19 patients sponsored by QuantumLeap Healthcare Collaborative. The I-SPY-COVID-19 Trial is investigating the clinical utility of cenicriviroc against macrophage attractant chemokine molecules designated CCR-2 and CCR-5 [40]. Preventing macrophages from gathering in alveoli would likely be most effective in the several days before SF levels rise. If the transformative event is thromboplastin-generated thrombin entering the pulmonary bloodstream, then that is the point at which anti-thromboplastin molecules will be most effective. Heparin [41] is one such agent as—in addition to its antithrombin effects—it releases tissue factor pathway inhibitor (TFPI), an inhibitor of the extrinsic clotting pathway. It also interferes with bradykinin generation by limiting the Factor XII-High Molecular Weight Kininogen interaction [42]. Other inhibitors of these para-coagulation pathways are known to exist.

If indeed, COVID-19 does co-opt clotting and has made itself substantially more lethal by so doing, it has at the same time made itself more vulnerable to therapeutic intervention.

## Data Availability

Not applicable.
